# Long-term exposure to road traffic noise and incidence of breast cancer: a cohort study

**DOI:** 10.1186/s13058-018-1047-2

**Published:** 2018-10-05

**Authors:** Zorana Jovanovic Andersen, Jeanette Therming Jørgensen, Lea Elsborg, Søren Nymand Lophaven, Claus Backalarz, Jens Elgaard Laursen, Torben Holm Pedersen, Mette Kildevæld Simonsen, Elvira Vaclavik Bräuner, Elsebeth Lynge

**Affiliations:** 1 Section of Environmental Health, Department of Public Health, University of Copenhagen, Øster Farimagsgade 5, 1014 Copenhagen, Denmark; 2 Nykøbing F Hospital, University of Copenhagen, Ejegodvej 63, 4800 Nykøbing F, Denmark; 3 DELTA Acoustics, Venlighedsvej 4, 2970 Hørsholm, Denmark; 4 Diakonissestiftelsen and Parker Institute, Frederiksberg Hospital, Peter Bangsvej 1, 2000 Frederiksberg, Denmark; 5 Juliane Marie Center, Department of Growth and Reproduction, Capital Region of Denmark, Rigshospitalet, Blegdamsvej 9, 2100 Copenhagen, Denmark

**Keywords:** Road traffic noise, Breast cancer, Estrogen receptor, Progesterone receptor, Nurses, Night shift work

## Abstract

**Background:**

Exposure to road traffic noise was associated with increased risk of estrogen receptor (ER)-negative (ER-) breast cancer in a previous cohort study, but not with overall or ER-positive (ER+) breast cancer, or breast cancer prognosis. We examined the association between long-term exposure to road traffic noise and incidence of breast cancer, overall and by ER and progesterone receptor (PR) status.

**Methods:**

We used the data from a nationwide Danish Nurse Cohort on 22,466 female nurses (age > 44 years) who at recruitment in 1993 or 1999 reported information on breast cancer risk factors. We obtained data on the incidence of breast cancer from the Danish Cancer Registry, and on breast cancer subtypes by ER and PR status from the Danish Breast Cancer Cooperative Group, up to 31 December 2012. Road traffic noise levels at the nurses’ residences were estimated by the Nord2000 method between 1970 and 2013 as annual means of a weighted 24 h average (L_den_) at the most exposed facade. We used time-varying Cox regression to analyze the associations between the 24-year, 10-year, and 1-year mean of L_den_ and breast cancer, separately for total breast cancer and by ER and PR status.

**Results:**

Of the 22,466 women, 1193 developed breast cancer in total during 353,775 person-years of follow up, of whom 611 had complete information on ER and PR status. For each 10 dB increase in 24-year mean noise levels at their residence, we found a statistically significant 10% (hazard ratio and 95% confidence interval 1.10; 1.00–1.20) increase in total breast cancer incidence and a 17% (1.17; 1.02–1.33) increase in analyses based on 611 breast cancer cases with complete ER and PR information. We found positive, statistically significant association between noise levels and ER+ (1.23; 1.06–1.43, *N* = 494) but not ER- (0.93; 0.70–1.25, *N* = 117) breast cancers, and a stronger association between noise levels and PR+ (1.21; 1.02–1.42, *N* = 393) than between noise levels and PR- (1.10; 0.89–1.37, *N* = 218) breast cancers. Association between noise and ER+ breast cancer was statistically significantly stronger in nurses working night shifts (3.36; 1.48–7.63) than in those not working at night (1.21; 1.02–1.43) (*p* value for interaction = 0.05).

**Conclusion:**

Long-term exposure to road traffic noise may increase risk of ER+ breast cancer.

**Electronic supplementary material:**

The online version of this article (10.1186/s13058-018-1047-2) contains supplementary material, which is available to authorized users.

## Background

Noise from road traffic is a persistent environmental stressor posing a huge and increasing health burden on urban populations. It was estimated in 2012 that environmental noise was responsible for at least one million healthy life years lost per year in Western Europe. [1] Epidemiological studies have shown that exposure to residential road traffic noise can lead to the development of cardiovascular disease and stroke [2], metabolic disease [3, 4], and possibly breast cancer (BC) [5–7].

The proposed mechanism behind the possible association between road traffic noise and BC include a psychological stress pathway, as persistent annoyance from exposure to environmental stressors such as traffic noise can lead to hyper-activation of the hypothalamic-pituitary-adrenal gland and release of stress hormones [8]. Accumulating evidence suggests that psychological stress increases the risk of BC, but the mechanism remains unknown [9]. Exposure to stress hormones (cortisol, catecholamines, etc.) can result in accumulation in DNA damage [10]. Stress hormone glucocorticoid steroid might promote tumor development and progression by inhibiting apoptosis [11]. In a single controlled experimental study in 18 healthy subjects, exposure to residential road traffic noise (48 or 75 dB) has been shown to result in increased levels of gene expression biomarkers of oxidative stress and DNA repair [12]. A recent experimental study found that rats exposed to noise (105 dB) for 30 days had significantly higher serum levels of malondialdehyde (MDA) and lower total antioxidant capacity (TAC), biomarkers of oxidative stress, than nonexposed rats [13]. Oxidative stress promotes BC development and progression [14, 15] and one study suggests that this mechanism is most relevant for estrogen receptor (ER)-positive (ER+) BC [15]. Another mechanism behind the possible link between noise exposure and BC involves sleep disturbance, reduced sleep quality and duration, which have been linked to residential road traffic noise exposure at night [16, 17]. Sleep disturbance and BC have been extensively studied with respect to night shift work, since “shift work that involves circadian disruption” was classified in 2007 as a probable human carcinogen by the International Agency for Research on Cancer (IARC) [18]. However, the epidemiological evidence on the relationship between night shift work and BC is mixed, as some meta-analyses suggest positive [19] and others no association [20, 21]. Similarly, the most recent literature on sleep duration and BC identifies no evidence of association [22, 23]. Finally, exposure to residential road traffic noise may increase the risk of weight gain [24], obesity [25, 26], and type II diabetes mellitus [27], all risk factors for postmenopausal BC [28, 29].

The evidence to date is mixed and there are three epidemiological studies on road traffic noise and BC, two on incidence [5, 7] and one on survival [6]. The study on long-term exposure to residential road traffic and railway noise and BC incidence in 29,875 women from the Danish Diet, Cancer and Health cohort detected a positive association between these exposures and ER-negative (ER-) BC, which comprises 20% of total BC, but found no association between exposure and ER+ or overall BC [5]. A study on BC survival in the same cohort found no association between residential road traffic noise and concurrent breast-cancer-specific mortality [6]. Finally, a case-control study of women living close to Frankfurt airport found no association between residential road traffic or railway noise and BC overall, but found a positive association between aircraft noise and ER- BC [7]. BCs classified by ER or PR expression have different clinical, pathologic, and molecular features and the etiology of these are heterogenous. Still, no study to date has investigated the association between traffic noise exposure and the incidence of BC classified by progesterone (PR) status.

Here we report on the association between exposure to residential road traffic noise over 24 years and the incidence of BC, overall and by subtypes, according to ER status, and for the first time, by PR status.

## Methods

### The Danish nurse cohort

The Danish Nurse Cohort [30] was inspired by the American Nurses’ Health Study to initially investigate the health effects of hormone therapy (HT) in a European population. The cohort was initiated in 1993 by sending a questionnaire to 23,170 female Danish nurses (age > 44 years), members of the Danish Nursing Organization, which included 95% of all nurses in Denmark. In total, 19,898 (86%) nurses replied, and the cohort was reinvestigated in 1999, including an additional 10,534 nurses who turned 45 years in the period 1993–1999 and 2231 non-responders from 1993, of whom 8833 in total (69%) replied. At recruitment, the nurses filled out a questionnaire on working conditions, weight and height, lifestyle (diet, active smoking, alcohol consumption, and leisure time physical activity), parity, age at first birth, age of menarche and menopause, and use of oral contraceptives (OC) and HT. We utilized baseline information from 1993 (19,898) or 1999 (8833) for 28,731 female nurses in total. Using a unique identification number, we linked the cohort participants to the Civil Registration System [31] to obtain vital status as of 31st December 2012 (active, date of death or emigration) and full residential address history since 1970.

### Breast cancer definition

We linked the records of 28,731 nurses using the unique identification number to the Danish Cancer Register [32] to extract all cancer diagnoses until the end of 2012. First, we identified nurses with diagnoses for any cancer (other than non-melanoma skin cancer) before baseline (1 April 1993 or 1 April 1999), and excluded these nurses from the analyses. Second, among nurses who were cancer-free at the cohort baseline, we extracted primary invasive BC diagnoses (ICD-10 codes C50), as the main outcome, and any other cancer (other than non-melanoma skin cancer), for censoring purposes, between cohort baseline (1 April 1993 or 1 April 1999) and 31 December 2012. We extracted data on BC subtype by ER and PR status from the clinical database of the Danish BC Cooperative Group [33], and in the subset of cases with available ER and PR status, we defined the following BC subtypes: ER+, ER-, PR+, PR-, ER+/PR+, ER+/PR-, ER-/PR+, and ER-/PR- BCs.

### Residential road traffic noise exposure

The road traffic noise levels at the nurses’ residential addresses were calculated using the Nord2000 method [34]. The Nord2000 method is the state-of-the-art traffic noise propagation model. It is based on input variables including geocodes of the location, the height of apartments above street level, road lines with information on yearly average daily traffic, traffic composition and speed, road type and properties (e.g. motorway, rural highway, road wider than 6 m, and other roads), building polygons for all surrounding buildings (height of buildings, etc.) and meteorology, including wind speed and direction, air temperature, and cloud cover. The traffic noise contribution is calculated for four weather classes, which typically occur in Denmark. The frequency of weather classes in the calculations are included with a frequency as they occur in a Danish meteorology average year. The propagation model is based on geometrical ray theory computing the 1/3 octave band sound attenuation along the path from the source to the receiver, accounting for the properties of the terrain (shape, ground type, including impedance and roughness) and variations in weather conditions, appropriate when estimating yearly average noise levels. Various weather conditions have been predefined and respective noise levels computed. The long-term noise levels, as the yearly average noise contributions, are then determined by weighting the occurrence of the different weather conditions obtained from weather statistics. The Nord2000 method has been validated by more than 500 propagation cases, 9 of them involving residential road traffic noise [35], and validation of the method has furthermore been conducted for noise originating from higher sources, e.g. wind turbines [36]. However, validation is not possible for historical values, and it is reasonable to assume that estimation of noise further back in time is less precise that that more recent. Annual average levels of residential road traffic noise were estimated for each nurse at each of her residential addresses between 1970 and 2013, as the equivalent continuous A-weighted sound pressure level (LA_eq_) at the most exposed façade of the dwelling for the day (L_d_, 0700–1900 h), evening (L_e_, 1900–2200 h) and night (L_n_, 2200–0700 h), and expressed as L_den_ (the overall noise level during the day, evening and night, calculated as the weighted 24-h noise level, with a 5 dB penalty for the noise levels in the evening hours, and a 10 dB penalty for the night time noise levels.). Our main noise exposure variable was the 24-year running mean of L_den_ from 1970 (oldest available) to 1993 or 1999, the beginning of study follow up. Additionally, we defined 10-year and 1-year running mean preceding diagnoses, and 1-year mean at the cohort baseline, to explore the effect of different exposure windows to residential road traffic noise. L_den_ was used also as a categorical variable with three levels, representing low (< 48 dB, 25th percentile of L_den_), medium (48–58 dB) and high (≥ 58 dB, 75th percentile of L_den_) residential road traffic noise exposure, for each time window. Finally, we explore the effect of LA_eq_, L_d_, L_e_, and L_n_, to explore whether day, evening, night, or overall exposure to residential road traffic noise was relevant for the risk of BC.

### Statistical analyses

We used an extended Cox proportional hazards regression model, with age as the underlying time scale, to examine the association between residential road traffic noise and incidence of overall BC in two steps: in a crude model adjusted for age (age as the underlying time scale), and in a fully adjusted model, where we additionally adjusted for birth cohort (1990–1934; 1935–1944; 1945–1949; 1950–1955), body mass index (BMI) (< 18.5 kg/m^2^; 18.5–25 kg/m^2^; 25–30 kg/m^2^; ≥ 30 kg/m^2^), alcohol use (none; moderate: 1–14 drinks/week; heavy: > 15 drinks/week), leisure time physical activity (low; medium; high), smoking status (never; former; current), age at menarche (years), parity (yes; no), number of children, age at first birth (years), menopausal status (yes; no), HT use (never; ever), and oral contraceptive (OC) use (never; ever). The start of follow up was age at the cohort baseline (1 April 1993 or 1st April 1999) and end of follow up was age at the time of BC diagnoses (event) diagnoses, other cancer diagnoses (except non-melanoma skin cancer), death, emigration, or 31 December 2012, whichever came first. We evaluated the effect of the residential road traffic noise as time-varying exposure, with 24-year, 10-year, and 1-year means calculated as geometric means, and modeled in separate models.

Sensitivity analyses were included using LA_eq_, L_d_, L_e_, and L_n_, and with checks for adherence to the proportional hazards assumption for all noise proxies and confounders based on scaled Schoenfeld residuals. The effect modification of an association between residential road traffic noise and BC by menopausal status, obesity, HT use, night shift work (yes - nurses those who were in work force at the cohort baseline and who reported typically working night shifts; no - nurses working at the cohort baseline and who typically work day, evening, or rotating shifts), and urbanicity (defined by population density at the municipality of residence at the cohort baseline in 1993 or 1999: rural areas - population density < 180 persons/km^2^; provincial areas with 180–5220 persons/km^2^; and urban areas with > 5220 persons/km^2^) was evaluated by introducing interaction terms into the Cox model, and was tested by the Wald test. Separate analyses were performed for subtypes of BC according to ER status (ER+ and ER-) PR status (PR+ and PR-) and ER status combined with PR status (ER+/PR+, ER+/PR-, ER-/PR- and ER-/PR+). Additional sensitivity analyses included analyses of association between 24-year mean L_den_ and overall BC with additional adjustment for the baseline year (year of recruitment 1993 or 1999), mean income at the municipality of residence at the cohort baseline, as a proxy of neighborhood socio-economic level, and air pollution, in terms of particulate matter (PM) less than 2.5 nm, (PM_2.5_) and nitrogen oxide (NO_x_) at the baseline year We did not adjust for air pollution in the main model, since air pollution is still not recognized as a risk factor for BC, and since we have previously found no association between air pollution and BC in this cohort [37]. Results were presented as hazard ratios (HRs) and 95% confidence intervals (CI). Analyses were performed using Stata 11.2.

## Results

Of the total 28,731 nurses in the Danish Nurse Cohort, we excluded 9 due to inactive (emigrated) vital status prior to study entry, 2556 with a cancer diagnosis before cohort baseline, 229 due to missing noise exposure, and 3471 with missing information on one or more covariates (see Additional file 1: Figure S1). Of the 22,466 nurses in the main analyses, 1193 developed BC during the mean follow up of 15.7 years or 353,775 person-years, with an incidence rate of 337 per 100,000 person-years. Of 1193 BC cases, information on ER status was available in 1061 cases and of these 884 (83.3%) were ER+ and 177 (16.7%) ER-. Of 1193 BC cases, information on both PR and ER status was available on 611 as follows: 393 (64.3%) were PR+ and 218 (35.7%) PR- and 494 (80.9%) were ER+ and 117 (19.1%) ER-. Among the 611 the combination of ER and PR status was that 384 (62.8%) were ER+/PR+, 110 (18.0%) ER+/PR-, 9 (1.5%) ER-/PR+, and 108 (17.8%) ER-/PR-.

**Fig. 1:**
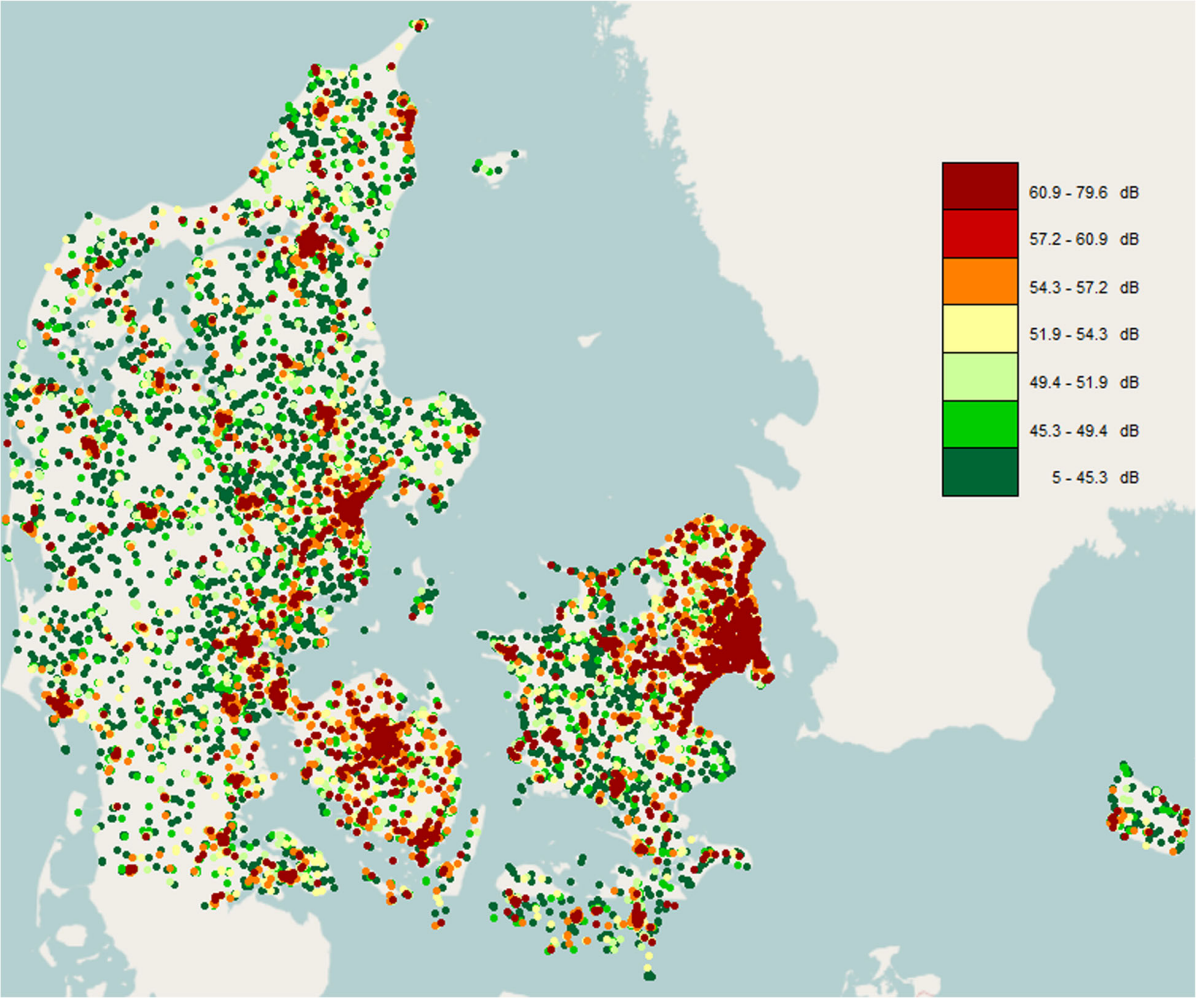
Mean residential road traffic noise levels (L_den_) at the year of cohort baseline (1993/99) among 22,466 members of the Danish Nurse Cohort in Denmark

The mean age at baseline was 53.0 years, mean BMI 23.7 kg/m^2^, 49.3% of the women were postmenopausal, 34.1% current smokers, 27.0% highly physically active, 22.8% heavy alcohol drinkers, 27.1% ever HT users, 58.9 ever OC users, and 14.1% were nulliparous and the mean age at 1st childbirth in parous women was 25.9 years (Table 1 compares baseline characteristics of non BC cases to all 1193 BC cases in the cohort, and Additional file 1: Table S1 compares baseline characteristics of non BC cases to 611 BC cases with complete date on ER and PR status). Compared with women who remained free of BC by the end of 2012, those who developed the cancer were more likely to be obese, current smokers, heavy alcohol drinkers, nulliparous, postmenopausal, older than 12 years at menarche, and HT users, but were less likely to be highly physically active and use OC. Mean level of residential road traffic noise at the year of the cohort baseline (1993 or 1999) was 48.6 dB and was slightly higher for women who developed BC and ranged from 5 dB to 79.6 dB as depicted in the geographical variation of residential road traffic noise in Fig. 1. As expected higher levels of traffic noise are found around major cities and roads.

**Table 1:** Descriptive statistics at the cohort baseline (1993 or 1999) among 22,466 female nurses from the Danish Nurse Cohort by breast cancer status at the end of follow up

	Total	Breast cancer	No breast cancer
	*N* = 22,466	*N* = 1193	*N* = 21,273
Age, mean (SD)	53.0 ± 7.9	53.6 ± 7.5	53.0 ± 7.9
Birth cohort			
< 1935, *n* (%)	5067 (22.6)	290 (24.3)	4777 (22.5)
1935–1944, *n* (%)	6878 (30.6)	446 (37.4)	6432 (30.2)
1945–1949, *n* (%)	4738 (21.1)	250 (21.0)	4488 (21.1)
≥ 1950, *n* (%)	5783 (25.7)	207 (17.4)	5576 (26.2)
Body mass index (BMI), mean (SD)	23.7 ± 3.5	23.8 ± 3.5	23.7 ± 3.5
BMI < 18.5 kg/m^2^, *n* (%)	544 (2.4)	24 (2.0)	520 (2.4)
BMI 18.5–24.9 kg/m^2^, *n* (%)	15,463 (68.8)	845 (70.8)	14,618 (68.7)
BMI 25–29.9 kg/m^2^, *n* (%)	5161 (23.0)	247 (20.7)	4914 (23.1)
BMI > 30 kg/m^2^, *n* (%)	1298 (5.8)	77 (6.5)	1221 (5.7)
Physical activity			
Low, *n* (%)	1466 (6.5)	79 (6.6)	1387 (6.5)
Medium, *n* (%)	14,944 (66.5)	806 (67.6)	14,138 (66.5)
High, *n* (%)	6056 (27.0)	308 (25.8)	5748 (27.0)
Smoking status			
Never, *n* (%)	7907 (35.2)	372 (31.2)	7535 (35.4)
Previous, *n* (%)	6901 (30.7)	356 (29.8)	6545 (30.8)
Current, *n* (%)	7658 (34.1)	465 (39.0)	7193 (33.8)
Alcohol consumption, mean (SD)	114.6 ± 128.1	123.2 ± 125.1	114.1 ± 128.2
Does not drink alcohol, *n* (%)	3444 (15.3)	189 (15.8)	3255 ± 15.3
Moderate drinker (1–14 drinks/week), *n* (%)	13,909 (61.9)	689 (57.8)	13,220 (62.1)
Heavy drinker (> 14 drinks/week), *n* (%)	5113 (22.8)	315 (26.4)	4798 (22.6)
Age at menarche			
≥ 12, *n* (%)	5431 (24.2)	301 (25.2)	5130 (24.1)
< 12, *n* (%)	17,035 (75.8)	892 (74.7)	16,143 (75.9)
Parity			
Nulliparous, *n* (%)	3165 (14.1)	192 (16.1)	2973 (14.0)
Parous, *n* (%)	19,301 (85.9)	1001 (83.9)	18,300 (86.0)
Number of births in parous women, mean (SD)	2.34 ± 0.88	2.31 ± 0.88	2.34 ± 0.88
Age at first birth, mean (SD)	25.9 ± 3.96	26.2 ± 4.11	25.9 ± 3.95
Menopausal status			
Premenopausal, *n* (%)	11,388 (50.7)	596 (50.0)	10,792 (50.7)
Post-menopausal, *n* (%)	11,078 (49.3)	597 (50.0)	10,481 (49.3)
Use of hormone therapy			
Never, *n* (%)	16,389 (73.0)	774 (64.9)	15,615 (73.4)
Previous, *n* (%)	2193 (9.8)	109 (9.1)	2084 (9.8)
Current, *n* (%)	3884 (17.3)	310 (26.0)	3574 (16.8)
Night work*			
Yes, *n* (%)	947 (5.4)	47 (5.1)	900 (5.4)
No, *n* (%)	16,598 (94.6)	873 (94.9)	15,725 (94.6)
Use of oral contraceptives			
Never, *n* (%)	9244 (41.1)	510 (42.7)	8734 (41.1)
Previous or current, *n* (%)	13,222 (58.9)	683 (57.3)	12,539 (58.9)
Residential area			
Urban, *n* (%)	3367 (15.0)	173 (14.5)	3194 (15.0)
Provincial, *n* (%)	9711 (43.2)	549 (46.0)	9162 (43.1)
Rural, *n* (%)	9388 (41.8)	471 (39.5)	8917 (41.9)
Road traffic noise levels at baseline residence			
L_den_, mean (SD)	52.7 ± 8.2	53.0 ± 8.1	52.7 ± 8.2
LA_24h_, mean (SD)	48.6 ± 8.2	48.9 ± 8.1	48.6 ± 8.2
L_d_, mean (SD)	50.4 ± 8.2	50.7 ± 8.2	50.4 ± 8.2
L_e_, mean (SD)	48.1 ± 8.1	48.4 ± 8.1	48.1 ± 8.1
L_n_, mean (SD)	44.6 ± 8.0	44.8 ± 8.0	44.5 ± 8.0

*Only available for 17,545 nurses

**Fig. 2:**
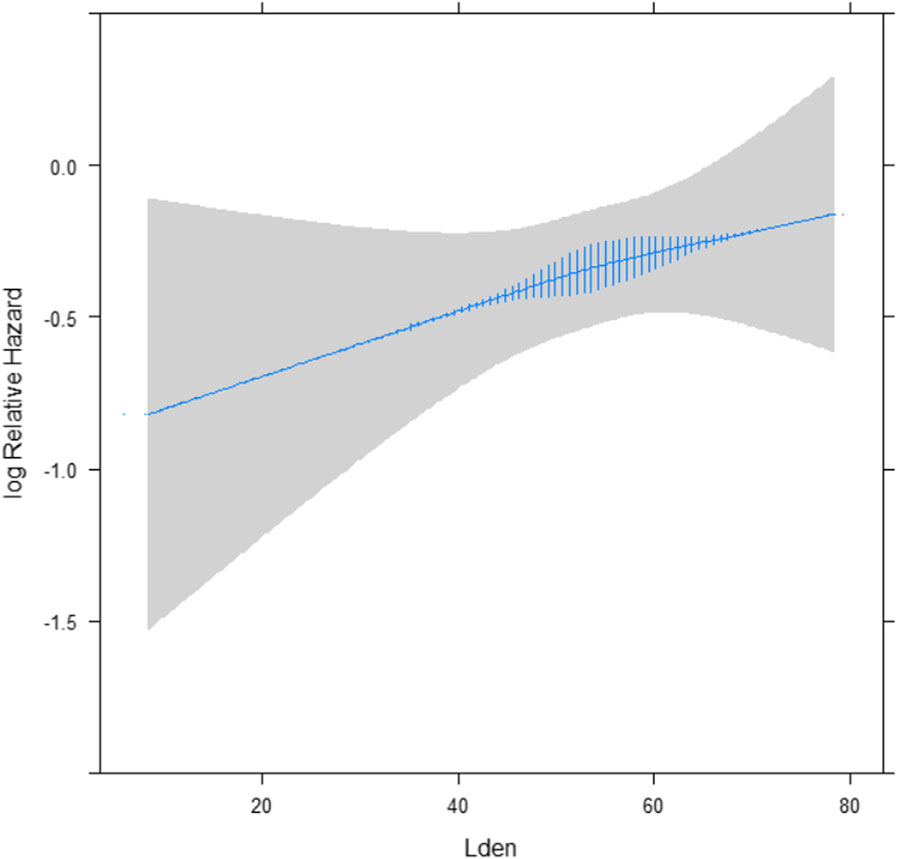
Association between residential exposure to road traffic noise level (L_den_) over 24 years and breast cancer (*N* = 1193) among 22,466 members of the Danish Nurse Cohort. Analyses adjusted for birth cohort, urbanization, body mass index, leisure time physical activity, alcohol consumption, age at menarche, parity, number of births, age at first birth, menopausal status, use of hormone therapy, use of oral contraceptives, and smoking status

In the fully adjusted models, we found a positive and statistically significant association between each 10 dB increase in residential road traffic noise levels at the residence (24-year mean noise levels preceding diagnosis) and incidence of BC, ranging from a 10% (HR; 95% CI, 1.10; 1.00–1.20) increase in total BC incidence (*N* = 1193), to a 17% (HR; 95% CI, 1.17; 1.02–1.33) increase in incidence based on the 611 BC cases with ER and PR information (Table 2). Figure 2 shows increasing HR with increase in time-weighted 24-year running mean preceding diagnosis based on the fully adjusted model and indicates a dose-response association. This dose response is also reflected in the fully adjusted models in Table 2; compared to women living in areas with low residential road traffic noise levels (< 48 dB), the highest HRs were observed in the fully adjusted models for women exposed to the highest noise levels (> 58 dB) (HR; 95% CI, 1.30; 1.07–1.60 in 1193 of all BC cases and 1.42; 1.06–1.89 in 611 BC cases with full ER and PR hormone receptor status); and smaller HRs in women exposed to medium noise levels (48–58 dB) (HR; 95% CI: 1.24; 1.04–1.47 in 1193 cases and 1.28; 0.99–1.65 in 611 cases). Similar results were observed with alternative categorization of L_den_ by quartiles of noise exposure (see Additional file 1: Table S8). The same trends, albeit weaker, were found with 10-year and 1-year mean noise levels preceding diagnosis. The weakest association was found with the 1-year mean levels at the cohort baseline (Table 2). All associations were statistically significant in analyses with the 611 BC cases with full information on estrogen and progesterone hormone status. Associations per 10 dB of the 24-year mean preceding diagnosis for LA_eq_, L_d_, L_e_, and L_n_ were almost identical to those with L_den_ (see Additional file 1: Table S2).

**Table 2:** Association between road traffic noise L_den_ and incidence of overall breast cancer in 22,466 nurses from Danish Nurse Cohort

Road traffic noise	All breast cancers *N* = 1193			Breast cancer with ER and PR status *N* = 611		
	Number of cases	Crude model^a^	Adjusted model^b^	Number of cases	Crude model^a^	Adjusted model^b^
		HR (95% CI)	HR (95% CI)		HR (95% CI)	HR (95% CI)
L_den_ 24 years preceding diagnoses						
Linear per 10 dB	1193	1.11 (1.02–1.21)*	1.10 (1.00–1.20)*	611	1.17 (1.04–1.31)*	1.17 (1.02–1.33)*
Low: < 48 dB	166	1.00	1.00	80	1.00	1.00
Medium 48–58 dB	675	1.24 (1.04–1.47)*	1.19 (1.00–1.42)*	352	1.35 (1.06–1.73)*	1.28 (0.99–1.65)
High > 58 dB	352	1.35 (1.12–1.62)*	1.30 (1.07–1.60)*	179	1.45 (1.11–1.89)*	1.42 (1.06–1.89)*
L_den_ 10 year preceding diagnoses						
Linear per 10 dB	1193	1.08 (1.00–1.16)*	1.06 (0.97–1.15)	611	1.16 (1.04–1.29)*	1.17 (1.04–1.32)*
Low < 48 dB	195	1.00	1.00	87	1.00	1.00
Medium 48–58 dB	646	1.19 (1.02–1.40)*	1.16 (0.98–1.37)	343	1.43 (1.13–1.81)*	1.38 (1.08–1.76)*
High > 58 dB	352	1.25 (1.05–1.49)*	1.21 (1.00–1.46)*	181	1.45 (1.13–1.88)*	1.45 (1.10–1.91)*
L_den_ 1 year at the year of diagnoses						
Linear per 10 dB	1192	1.08 (1.01–1.17)*	1.07 (0.99–1.16)	610	1.15 (1.04–1.27)*	1.16 (1.04–1.30)*
Low < 48 dB	202	1.00	1.00	98	1.00	1.00
Medium 48–58 dB	644	1.25 (1.06–1.46)*	1.22 (1.03–1.43)*	333	1.34 (1.07–1.68)*	1.29 (1.03–1.63)*
High > 58 dB	346	1.29 (1.09–1.54)*	1.26 (1.04–1.52)*	179	1.39 (1.09–1.78)*	1.39 (1.07–1.81)*
L_den_ 1 year at the baseline (1993/99)						
Linear per 10 dB	1193	1.05 (.98–1.13)	1.04 (0.96–1.12)	611	1.11 (1.01–1.23)*	1.12 (1.00–1.26)*
Low < 48 dB	235	1.00	1.00	110	1.00	1.00
Medium 48–58 dB	648	1.17 (1.01–1.36)*	1.13 (0.97–1.33)	347	1.34 (1.08–1.67)*	1.29 (1.03–1.62)*
High > 58 dB	310	1.20 (1.01–1.42)*	1.17 0.97–1.41)	154	1.29 (1.01–1.64)*	1.29 (0.98–1.69)

*Abbreviations*: *ER* Estrogen receptor, *PR* Progesterone receptor

^a^Crude model with age as underlying time scale

^b^Model adjusted for birth cohort, urbanization (urban, provincial, rural), body mass index (underweight, normal, overweight, obese), leisure time physical activity (low, medium, high), alcohol consumption (low, moderate, heavy), age at menarche (≤ 12 years of age, > 12 years of age), parity (nulliparous, parous), number of births, age at first birth, menopausal status (premenopausal, postmenopausal), use of hormone therapy (never, past, current), use of oral contraceptives (never, ever), and smoking status

*p-value<0.05

We found a positive and statistically significant association between residential road traffic noise (for each 10 dB increase in 24-year mean noise levels preceding diagnosis) and ER+ (1.23; 1.06–1.43, *N* = 494) and none with ER- (HR; 95% CI, 0.93; 0.70–1.25, *N* = 117) BCs (Table 3). The association with PR+ BC was positive and statistically significant (HR; 95% CI, 1.21; 1.02–1.42, *N* = 393), and the association with PR- BC (HR; 95% CI, 1.10; 0.89–1.37, *N* = 218) was positive, albeit statistically non-significant. Compared to women exposed to noise levels < 48 dB, women exposed to noise levels > 58 dB had 59% (HR; 95% CI, 1.59; 1.14–2.20) and 66% (HR; 95% CI, 1.66; 1.14–2.40) higher risk of developing ER+ and PR+ BC, respectively. Results were consistent in a sample of 1061 BC cases with data on ER status, but not PR status (see Additional file 1: Table S3).

**Table 3:** Association^a^ between road traffic noise L_den_ and incidence of breast cancer by ER and PR status in 22,466 nurses with estrogen and progesterone hormone receptor status from the Danish Nurse Cohort

	Breast cancer type by ER status				Breast cancer type by PR status			
	Number of cases	ER+	Number of cases	ER-	Number of cases	PR+	Number of cases	PR-
		HR (95% CI)		HR (95% CI)		HR (95% CI)		HR (95% CI)
24 years preceding diagnosis								
Linear per 10 dB	494	1.23 (1.06–1.43)*	117	0.93 (0.70–1.25)	393	1.21 (1.02–1.42)*	218	1.10 (0.89–1.37)
Low < 48 dB	58	1.00	22	1.00	45	1.00	35	1.00
Medium 48–58 dB	290	1.48 (1.10–1.98)*	62	0.77 (0.46–1.28)	231	1.51 (1.08–2.10)*	121	0.99 (0.67–1.47)
High > 58 dB	146	1.59 (1.14–2.20)*	33	0.97 (0.54–1.75)	117	1.66 (1.14–2.40)*	62	1.11 (0.70–1.75)
10 years preceding diagnosis								
Linear per 10 dB	494	1.22 (1.07–1.40)*	117	1.00 (0.76–1.30)	393	1.20 (1.03–1.40)*	218	1.13 (0.92–1.37)
Low < 48 dB	64	1.00	23	1.00	51	1.00	36	1.00
Medium 48–58 dB	279	1.55 (1.17–2.05)*	64	0.90 (0.54–1.48)	223	1.55 (1.13–2.12)*	120	1.13 (0.77–1.67)
High > 58 dB	151	1.65 (1.20–2.26)*	30	0.89 (0.49–1.61)	119	1.65 (1.16–2.35)*	62	1.16 (0.74–1.81)
1 year at the year of diagnosis								
Linear per 10 dB	494	1.18 (1.04–1.33)*	116	1.10 (0.85–1.42)	392	1.21 (1.05–1.39)*	218	1.08 (0.90–1.31)
Low < 48 dB	77	1.00	21	1.00	59	1.00	39	1.00
Medium 48–58 dB	268	1.34 (1.03–1.74)*	65	1.13 (0.68–1.89)	216	1.42 (1.05–1.91)*	117	1.11 (0.76–1.62)
High > 58 dB	149	1.47 (1.09–1.98)*	30	1.12 (0.61–2.03)	117	1.53 (1.09–2.15)*	62	1.18 (0.76–1.82)

*Abbreviations*: *ER* Estrogen receptor, *PR* Progesterone receptor

^a^Model adjusted for birth cohort, urbanization (urban, provincial, rural), body mass index (underweight, normal, overweight, obese), leisure time physical activity (low, medium, high), alcohol consumption (low, moderate, heavy), age at menarche (≤ 12 years of age, > 12 years of age), parity (nulliparous, parous), number of births, age at first birth, menopausal status (premenopausal, postmenopausal), use of hormone therapy (never, past, current), use of oral contraceptives (never, ever) and smoking status

*p value <0.05

When considering BC per combined estrogen and progesterone status, we found the strongest associations between residential road traffic noise (for each 10 dB increase in 24-year mean noise levels preceding diagnosis) and ER+/PR+ (HR; 95% CI, 1.22; 1.02–1.42, *N* = 384) and ER+/PR- (HR; 95% CI, 1.33; 0.97–1.82, *N* = 110), and none with ER-/PR+ (*N* = 9) or ER-/PR- (*N* = 108) BCs (Table 4). Descriptive statistics for nurses by combination of ER and PR status are given in Additional file 1: Table S4.

**Table 4:** Association^a^ between road traffic noise L_den_ and incidence of breast cancer by combination of estrogen and progesterone receptor status in 22,466 nurses from Danish Nurse Cohort

	ER+/PR+		ER+/PR-		ER-/PR+		ER-/PR-	
	Number of cases	HR (95% CI)	Number of cases	HR (95% CI)	Number of cases	HR (95% CI)	Number of cases	HR (95% CI)
24 years preceding diagnosis								
Linear per 10 dB	384	1.21 (1.02–1.42)*	110	1.33 (0.97–1.82)	9	1.14 (0.33–3.87)	108	0.92 (0.68–1.25)
Low < 48 dB	45	1.00	13	1.00	0	1.00	22	1.00
Medium 48–58 dB	226	1.49 (1.07–2.07)*	64	1.45 (0.78–2.69)	5	–	57	0.73 (0.43–1.22)
High > 58 dB	113	1.61 (1.11–2.34)*	33	1.51 (0.75–3.03)	4	–	29	0.88 (0.48–1.62)
10 years preceding diagnosis								
Linear per 10 dB	384	1.20 (1.03–1.40)*	110	1.30 (0.98–1.74)	9	1.22 (0.40–3.72)	108	0.98 (0.74–1.29)
Low < 48 dB	51	1.00	13	1.00	0	1.00	23	1.00
Medium 48–58 dB	218	1.53 (1.11–2.09)*	61	1.65 (0.89–3.06)	5	–	59	0.85 (0.51–1.40)
High > 58 dB	115	1.61 (1.13–2.29)*	36	1.83 (0.92–3.62)	4	–	26	0.79 (0.43–1.46)
1 year preceding diagnosis								
Linear per 10 dB	384	1.21 (1.05–1.39)*	110	1.07 (0.83–1.40)	8	1.15 (0.39–3.40)	108	1.10 (0.84–1.43)
Low < 48 dB	58	1.00	19	1.00	1	1.00	20	1.00
Medium 48–58 dB	212	1.42 (1.05–1.92)*	56	1.09 (0.64–1.87)	4	1.02 (0.11–9.59)	61	1.14 (0.67–1.92)
High > 58 dB	114	1.53 (1.09–2.15)*	35	1.27 (0.69–2.34)	3	1.35 (0.13–14.4)	27	1.09 (0.59–2.03)

*Abbreviations*: *ER* Estrogen receptor, *PR* Progesterone receptor

^a^Model adjusted for birth cohort, urbanization (urban, provincial, rural), body mass index (underweight, normal, overweight, obese), leisure time physical activity (low, medium, high), alcohol consumption (low, moderate, heavy), age at menarche (≤ years of age, > 12 years of age), parity (nulliparous, parous), number of births, age at first birth, menopausal status (premenopausal, postmenopausal), use of hormone therapy (never, past, current), use of oral contraceptives (never, ever) and smoking status

*p value <0.05

There was no effect modification of association between residential road traffic noise and BC in which estrogen and progesterone hormone receptor status was available (*N* = 611), in ER+, PR+, or ER/PR+ BCs by menopausal status, HT use, obesity, or residential area. (Table 5). Results were similar for the 1193 BC cases (Additional file 1: Table S5). We did, however, find a statistically significantly (*p* value for interaction = 0.05) stronger association between residential road traffic noise (for each 10 dB increase in 24-year mean noise levels) and ER+ BC in nurses working night shifts (HR; 95% CI, 3.36; 1.48–7.63) than in those not working at night (HR; 95% CI, 1.21; 1.02–1.43) (Table 5).

**Table 5:** Modifications of association^a^ between 24-year mean road traffic noise L_den_ (per 10 dB) and incidence of breast cancer by menopausal status, HT use, obesity, night work, and residential area in 22,466 nurses from the Danish Nurse Cohort

	Breast cancer with receptor status *N* = 611		ER+ *N* = 494		PR+ *N* = 393		ER+/PR+ *N* = 384	
	Number of cases	HR (95% CI)	Number of cases	HR (95% CI)	Number of cases	HR (95% CI)	Number of cases	HR (95% CI)
Menopausal status								
Premenopausal	334	1.16 (0.97–1.38)	272	1.25 (1.02–1.52)	231	1.17 (0.95–1.45)	226	1.19 (0.96–1.48)
Postmenopausal	277	1.18 (0.97–1.43)	222	1.21 (0.97–1.51)	162	1.26 (0.97–1.63)	158	1.23 (0.94–1.59)
*p* value for interaction^**^		0.9069		0.7135		0.6767		0.8909
HT use								
Never	390	1.16 (0.99–1.36)	319	1.21 (1.01–1.45)	256	1.18 (0.97–1.44)	254	1.19 (0.97–1.45)
Previous	57	0.98 (0.64–1.50)	44	1.12 (0.68–1.85)	33	1.03 (0.58–1.84)	31	0.99 (0.55–1.80)
Current	164	1.26 (0.96–1.65)	131	1.33 (0.99–1.80)	104	1.34 (0.96–1.88)	99	1.34 (0.95–1.89)
*p* value for interaction^**^		0.4685		0.6047		0.5697		0.6012
Obesity (BMI > 30 kg/m^2^)								
No	572	1.18 (1.03–1.35)	459	1.25 (1.07–1.45)	367	1.22 (1.03–1.45)	358	1.22 (1.03–1.46)
Yes	39	1.03 (0.65–1.65)	35	1.04 (0.63–1.71)	26	1.03 (0.59–1.78)	26	1.03 (0.59–1.78)
*p* value for interaction^**^		0.9805		0.9489		0.7553		0.7704
Night work*								
No	473	1.16 (1.00–1.35)	386	1.21 (1.02–1.43)	317	1.22 (1.02–1.47)		1.23 (1.02–1.48)
Yes	24	1.86 (0.97–3.57)	17	3.36 (1.48–7.63)	9	1.88 (0.59–6.00)	310	3.04 (0.80–11.60)
*p* value for interaction^**^		0.3754		0.0477*		0.3000	8	0.1585
Residential area								
Urban	77	1.30 (0.81–2.10)	67	1.24 (0.74–2.07)	50	0.95 (0.52–1.75)	49	0.96 (0.52–1.77)
Provincial	299	1.21 (0.98–1.51)	234	1.43 (1.12–1.83)	192	1.57 (1.19–2.06)	185	1.57 (1.19–2.08)
Rural	235	1.12 (0.94–1.33)	193	1.12 (0.92–1.35)	151	1.06 (0.85–1.31)	150	1.06 (0.86–1.32)
*p* value for interaction^**^		0.6460		0.2610		0.0811		0.0893

^a^Model adjusted for birth cohort, urbanization (urban, provincial, rural), body mass index (underweight, normal, overweight, obese), leisure time physical activity (low, medium, high), alcohol consumption (low, moderate, heavy), age at menarche (≤ 12 years of age, > 12 years of age), parity (nulliparous, parous), number of births, age at first birth, menopausal status (premenopausal, postmenopausal), use of hormone therapy (never, past, current), use of oral contraceptives (never, ever) and smoking status

Traffic noise was entered as a continuous variable in all models as the 24-year running mean preceding diagnosis. Model adjusted for birth cohort, urbanization (urban, provincial, rural), body mass index (underweight, normal, overweight, obese), leisure time physical activity (low, medium, high), alcohol consumption (low, moderate, heavy), age at menarche (≤ years of age, > 12 years of age), parity (nulliparous, parous), number of births, age at first birth, menopausal status (premenopausal, postmenopausal), use of hormone therapy (never, past, current), use of oral contraceptives (never, ever) and smoking status. However, there was no adjustment for the stratification variable

*HT* hormone therapy, *BMI* body mass index, *ER* estrogen receptor, *PR* progesterone receptor

*Only available for 17,545 nurses

^**^Test of the null hypothesis that the linear trends are identical, for Wald’s test for interaction

We found that our results were robust to additional adjustment for the baseline year (1993 or 1999) and mean municipality income (Additional file 1: Table S6). Similarly, the main results were unchanged when adjusting for air pollution (Additional file 1: Table S7).

## Discussion

We detected an association between road traffic noise levels at residences and BC incidence. The association was strongest for ER+ and PR+ BCs, and no association was found for ER- or PR- BCs. Nurses working at night may be more susceptible to the adverse effects of noise.

We present a novel finding of association between residential road traffic noise and BC overall, as well with ER+ and PR+ BC subtypes, in contrast to findings from two existing studies [5, 7]. Sørensen et al. linked road traffic and railway noise levels at residences to the postmenopausal BC incidence in 29,875 women from Danish Diet, Cancer and Health cohort, recruited between 1993 and 1997 (age 50–65 years) and found no association with total (HR; 95% CI, 1.02; 0.93–1.11) or with ER+ (HR; 95% CI, 0.99: 0.90–1.10) BCs, for each increase of 10 dB in 10-year mean noise levels, the longest available exposure window in that study [5]. However, the authors found 28%, 23% and 20% increases in ER- BC incidence per 10 dB increase in 1-year-, 5-year, and 10-year mean residential road traffic noise levels, respectively, suggesting the recent noise levels to be more relevant than the accumulated levels over many years [5]. In the Danish Nurse Cohort we found that the long-term exposure over 24 years was most relevant for the risk of BC, and that this association was strongest for the ER+ BCs (Table 3 and Table 4). Interestingly, although we found no association between long-term noise exposure and ER- BC incidence, a positive, association was found with the most recent 1-year exposure window (HR; 95% CI, 1.10; 0.85–1.42) (Table 3). These results could suggest that early life and historical, long-term exposures to noise are most relevant for ER+ BC, while the more recent exposure to noise may be important for ER- BCs.

Several factors may explain discrepancies in results between the two Danish studies. First, the population age varied; while we included all BCs in women age > 44 years, including premenopausal and postmenopausal cancers, Sørensen et al. focused on an older population of postmenopausal women, age > 55 years [5]. Second, the geographical location of the residences differs between the cohorts, as the Sørensen et al. study included women only from highly urban areas (Copenhagen and Aarhus) whereas the present study included nurses from the whole of Denmark, residing primarily in rural (42%) and provincial (43%) areas (Fig. 1). These factors seem not to explain the differences between two studies, as we found no evidence of effect modification by menopausal status or urbanicity (Table 5 and Additional file 1: Table S5). Third, the method of modeling residential road traffic noise and the number of years of follow up varied between the two studies; in the present study we used a state-of-the art Nord200 noise model providing annual mean estimates of residential road traffic noise at the nurses’ addresses, allowing modeling of time-varying effects of noise exposure going back as far as 24 years, the longest exposure window to date. This model is considered superior to the Soundplan model used in the Sørensen et al. study, which estimates residential road traffic noise data at a lower resolution as 5-year averages, and was only available at 10 years prior to BC diagnoses in the study of Sørensen et al. [5]. Thus, varying ability of the available noise data to better capture early exposure to residential road traffic noise, which may be more relevant for ER+ BCs, may explain the differences in our results compared to those of Sørensen et al. [5].

Hegewald et al., in a case-control (6643 cases and 471,596 controls) study of women older than 40 years and living close to Frankfurt airport between 2006 and 2010, linked residential road traffic noise data from 2005 to BC incidence, in total, and by ER status [7]. They found no association between residential road traffic noise (for each 10 dB increase) and total BC risk (odds ratio (OR); 85% CI, 0.99; 0.96–1.02), ER+ (OR; 95% CI, 0.98; 0.95–1.02), or ER- (OR; 95% CI 1.01; 0.96–1.07) [7]. Residential road traffic noise levels and geographical distribution of that cohort were comparable to ours ranging from < 40 dB to 85.7 dB, but the noise level was estimated for 2005 only, thus representing only recent exposure. Furthermore, Hegewald et al. used prescriptions of anti-estrogens or aromatase inhibitors as indicators of ER+ tumors, by which they have likely underestimated the number of ER+ BCs (69.9%) and overestimated the number of ER- (30.1%) BCs. Both Danish studies used clinical data on ER and PR status. These inconsistencies in the literature call for more studies on residential road traffic noise and BC incidence.

We present novel finding of increased susceptibility to residential road traffic noise in nurses who work at night, as compared to those who typically work day, evening or rotating shifts (Table 5). This may be explained by smaller exposure misclassification, as nurses who work at night are at home in the daytime, when residential road traffic noise levels are highest, and when their day sleep or daily activities are more likely to be disrupted by noise, and result in annoyance and health effects. Disruptions to circadian rhythms due to night shift work have been shown to contribute to endocrine-dependent diseases, including breast carcinogenesis, by negatively impacting neuroendocrine and neuroimmune cells [38]. Our finding thus may suggest that women with circadian rhythm disruptions may be more susceptible to effects of noise than those without. Another possible explanation is that night shift workers represent a sensitive group, as they already have increased risk of poor sleep, sleep disturbance, lack of sleep, work-related stress, fatigue, etc. Sensitivity appears to play a key part in the health effects of environmental noise, as a specific type and level of noise may interact with sensitivity, causing some degree of annoyance and physiological response [4]. Although data on susceptibility to noise effects related to BC are sparse, studies with other health outcomes have suggested that the health effects of noise are enhanced and possibly limited to those who are annoyed by noise or susceptible to the effects of noise. For example, the association between aircraft or residential road traffic noise and increased hypertension has been limited only to those who reported annoyance by noise [2]. Similarly, a study found an association between residential road traffic noise and depressive symptoms only in those who suffer from insomnia [39], and an association between residential road traffic noise and markers of obesity have been detected only in highly noise-sensitive women [25].

We present novel findings of the relevance of residential road traffic noise in ER+ but not in ER- BCs. Our finding are similar to the findings of the recent study, suggesting that the oxidative stress pathway promoting BC development and progression [12] was most relevant in ER+ BC [15]. Residential road traffic noise has been linked to increased risk of weight gain [24] and obesity [25] and BMI has been positively associated with risk of ER+ postmenopausal BC, but not with ER- BC [40]. It has been also suggested that lack of melatonin, due to sleep disturbance and light exposure at night, may be related to increased risk of ER+ breast cancer, although reports in the literature are not completely consistent [41].

The strength of this study include having data from a large, prospective nationwide cohort of 22,466 women residing in rural, provincial, and urban areas, providing for large contrasts in residential road traffic noise levels (Fig. 1). We benefited from having access to data from well-established Danish clinical cancer registers with detailed and validated information on BC incidence and subtypes by ER status, and for the first time, by PR status. We benefited from well-defined information on all relevant risk factors for BC, and associations between BC and established risk factors, such as alcohol use [42], smoking [43], and HT [44, 45] have been documented earlier in this cohort. The cohort consists of a rather homogenous population of female nurses, with similar education, occupation, and socioeconomic status, minimizing the possibility of residual confounding by these factors. Danish nurses have been found to have a generally healthier lifestyle than a representative sample of Danish women, as they smoked less and had higher physical activity levels, but consumed more alcohol [30]. There are no large differences between Danish nurses and Danish women in general in the use of health care and in disease occurrence [30]. It is therefore reasonable to generalize the findings based on the Danish Nurses Cohort to Danish, and other, women in general.

We also benefited from having information on air pollution exposure in this cohort, an important confounder since air pollution and road traffic noise share the same major source (traffic) and are highly correlated. We have, however, found that additional adjustment for air pollution did not affect reported associations between road traffic noise and BC, which is line with our previous study in this cohort where we found no association between air pollution and BC [37]. We benefitted from a state-of-the art Nord200 noise model providing historical annual mean estimates of residential road traffic noise at the nurses’ addresses, allowing for modeling of time-varying effects of noise exposure going back as far as 24 years and providing the longest exposure window to date. The weakness of the study is lack of information on annoyance from noise, noise from neighbors, social noise, hearing impairment, noise exposure at work, time-activity patterns and time spent at home, placement of the bedroom, and window opening habits, etc. Modeled levels of noise at the most exposed façade at home are thus inherently associated with a certain levels of exposure misclassification and deviation from real personal exposures to noise. However, if this misclassification is non-differential, and effect estimates are likely biased towards zero.

## Conclusions

In this large cohort of Danish female nurses older than 44 years, we found a positive association between residential road traffic noise and risk of BC. We present two novel findings: the association seemed limited to ER+ BCs, and nurses working at night may be especially susceptible to the adverse effects of noise. Our findings are in contrast to earlier finding of an association between road traffic noise and ER- BC [5], calling urgently for more data on noise and BC.

## Additional file

Additional file 1: **Table S1.** Descriptive statistics at the cohort baseline (1993 or 1999) among 22,466 female nurses from the Danish Nurse Cohort by BC status at the end of follow up. **Table S2.** Association between 24-year mean road traffic noise L_den_, LA_eq_, L_d_, L_e_, and L_n_, and incidence of overall BC and BC in women with receptor status available, among 22,466 nurses from the Danish Nurse Cohort. **Table S3.** Association between road traffic noise L_den_ and incidence of BC by ER and PR status in 22,466 nurses from the Danish Nurse Cohort. **Table S4.** Descriptive statistics at the year of cohort baseline (1993 or 1999) among 21,884 members of the Danish Nurse Cohort, by estrogen and progesterone BC receptor status. **Table S5.** Modifications of association^a^ between 24-year mean road traffic noise L_den_ (per 10 dB) and incidence of BC by menopausal status, hormone therapy (HT) use, obesity, night work, and residential area in 22,466 nurses from the Danish Nurse Cohort. **Table S6.** Association between road traffic noise L_den_ (24 years preceding diagnosis) and incidence of overall BC in nurses from the Danish Nurse Cohort with additional adjustments for year of cohort inclusion (1993 or 1999) and average municipality income. **Table S7.** Association between road traffic noise L_den_ (24 years preceding diagnosis) and incidence of overall BC in nurses from the Danish Nurse Cohort with additional adjustments for air pollutants. **Table S8.** Association between road traffic noise L_den_ and incidence of overall BC in nurses from the Danish Nurse Cohort with additional noise categories, defined from quartiles of residential road traffic noise exposure at the year of cohort inclusion. **Table S9.** Descriptive statistics at the cohort baseline (1993 or 1999) among 1193 female nurses from the Danish Nurse Cohort with BC status at the end of follow up. **Figure S1.** Flow chart of inclusion criteria and the study population. (DOCX 101 kb)

## Abbreviations

BC: Breast cancer
BMI: Body mass index
CI: Confidence interval
dB: Decibel
ER: Estrogen receptor
HR: Hazard ratio
HT: Hormone therapy
IARC: International Agency for Research on Cancer
LA_eq_: A-weighted sound pressure level
L_d_: A-weighted sound pressure level during the day 0700–1900 h
L_den_: Overall A-weighted sound pressure level during the day, evening, and night
L_e_: A-weighted sound pressure level during the evening 1900–2200 h
L_n_: A-weighted sound pressure level during the night 2200–0700 h
OC: Oral contraceptives
PR: Progesterone receptor
