# Supplementary material for: Long-term exposure to road traffic noise and incidence of breast cancer: a cohort study
Source: Breast Cancer Res. 2018 Oct 5;20:119. doi: 10.1186/s13058-018-1047-2 (PMC6173937; doi:10.1186/s13058-018-1047-2)
Supplement: Supplementary file 1 — Table S1. Descriptive statistics at the cohort baseline (1993 or 1999) among 22,466 female nurses from the Danish Nurse Cohort by BC status at the end of follow up. Table S2. Association between 24-year mean road traffic noise Lden, LAeq, Ld, Le, and Ln, and incidence of overall BC and BC in women with receptor status available, among 22,466 nurses from the Danish Nurse Cohort. Table S3. Association between road traffic noise Lden and incidence of BC by ER and PR status in 22,466 nurses from the Danish Nurse Cohort. Table S4. Descriptive statistics at the year of cohort baseline (1993 or 1999) among 21,884 members of the Danish Nurse Cohort, by estrogen and progesterone BC receptor status. Table S5. Modifications of associationa between 24-year mean road traffic noise Lden (per 10 dB) and incidence of BC by menopausal status, hormone therapy (HT) use, obesity, night work, and residential area in 22,466 nurses from the Danish Nurse Cohort. Table S6. Association between road traffic noise Lden (24 years preceding diagnosis) and incidence of overall BC in nurses from the Danish Nurse Cohort with additional adjustments for year of cohort inclusion (1993 or 1999) and average municipality income. Table S7. Association between road traffic noise Lden (24 years preceding diagnosis) and incidence of overall BC in nurses from the Danish Nurse Cohort with additional adjustments for air pollutants. Table S8. Association between road traffic noise Lden and incidence of overall BC in nurses from the Danish Nurse Cohort with additional noise categories, defined from quartiles of residential road traffic noise exposure at the year of cohort inclusion. Table S9. Descriptive statistics at the cohort baseline (1993 or 1999) among 1193 female nurses from the Danish Nurse Cohort with BC status at the end of follow up. Figure S1. Flow chart of inclusion criteria and the study population. (DOCX 101 kb) [file 13058_2018_1047_MOESM1_ESM.docx]

**Additional file 1**

Table S1. Descriptive statistics at the cohort baseline (1993 or 1999) among 22,466 female nurses from the Danish Nurse Cohort by breast cancer status at the end of follow-up.

|  | Total | Breast cancer* | No breast cancer |
| --- | --- | --- | --- |
|  | N = 22,466 | N = 611 | N = 21,273 |
| **Age, mean ± SD** | 53.0 ± 7.9 | 52.8 ± 7.0 | 53.0 ± 7.9 |
| **Birth cohort** |  |  |  |
| <1935, n (%) | 5,067 (22.6) | 134 (21.9) | 4,777 (22.5) |
| 1935-1944, n (%) | 6,878 (30.6) | 216 (35.4) | 6,432 (30.2) |
| 1945-1949, n (%) | 4,738 (21.1) | 133 (21.8) | 4,488 (21.1) |
| ≥ 1950, n (%) | 5,783 (25.7) | 128 (20.9) | 5,576 (26.2) |
| **Body Mass Index, mean ± SD** | 23.7 ± 3.5 | 23.9 ± 3.4 | 23.7 ± 3.5 |
| BMI <18.5 kg/m^2^, n (%) | 544 (2.4) | 9 (1.5) | 520 (2.4) |
| BMI 18.5-24,9 kg/m^2^, n (%) | 15,463 (68.8) | 439 (71.8) | 14,618 (68.7) |
| BMI 25-29.9 kg/m^2^, n (%) | 5,161 (23.0) | 124 (20.3) | 4,914 (23.1) |
| BMI >30 , n (%) | 1,298 (5.8) | 39 (6.4) | 1,221 (5.7) |
| **Physical activity** |  |  |  |
| Low, n (%) | 1,466 (6.5) | 38 (6.2) | 1,387 (6.5) |
| Medium, n (%) | 14,944 (66.5) | 406 (66.4) | 14,138 (66.5) |
| High, n (%) | 6,056 (27.0) | 167 (27.3) | 5,748 (27.0) |
| **Smoking status** |  |  |  |
| Never, n (%) | 7,907 (35.2) | 182 (29.8) | 7,535 (35.4) |
| Previous, n (%) | 6,901 (30.7) | 193 (31.6) | 7,193 (33.8) |
| Current, n (%) | 7,658 (34.1) | 236 (38.6) | 6,545 (30.8) |
| **Alcohol consumption, mean ± SD** | 114.6 ± 128.1 |  | 114.1 (128.2) |
| Does not drink alcohol, n (%) | 3,444 (15.3) | 85 (13.9) | 3,255 (15.3) |
| Moderate drinker (1-14 drinks/week), n (%) | 13,909 (61.9) | 372 (60.9) | 13,220 (62.1) |
| Heavy drinker (> 14 drinks/week), n (%) | 5,113 (22.8) | 154 (25.2) | 4,798 (22.6) |
| **Age at menarche (years)** |  |  |  |
| ≥ 12, n (%) | 5431 (24.2) | 159 (26.0) | 5,130 (24.1) |
| <12, n (%) | 17,035 (75.8) | 452 (74.0) | 16,143 (75.9) |
| **Parity** |  |  |  |
| Nulliparous, n (%) | 3,165 (14.1) | 90 (14.7) | 2,973 (14.0) |
| Parous, n (%) | 19,301 (85.9) | 521 (85.3) | 18,300 (86.0) |
| **Number of births in parous women, mean ± SD** | 2.34 ± 0.88 | 2.3 ± 0.85 | 2.34 ± 0.88 |
| **Age at first birth in parous women, mean ± SD** | 25.9 ± 3.96 | 26.1 ± 4.10 | 25.9 ± 3.95 |
| **Menopausal status** |  |  |  |
| Pre-menopausal, n (%) | 11,388 (50.7) | 334 (54.7) | 10,792 (50.7) |
| Post-menopausal, n (%) | 11,078 (49.3) | 277 (45.3) | 10,481 (49.3) |
| **Use of hormone therapy (HT)** |  |  |  |
| Never, n (%) | 16,389 (73.0) | 390 (63.8) | 15,615 (73.4) |
| Previous, n (%) | 2,193 (9.8) | 57 (9.3) | 2,084 (9.8) |
| Current, n (%) | 3,884 (17.3) | 164 (26.8) | 3,574 (16.8) |
| **Use of oral contraceptives** |  |  |  |
| Never, n (%) | 9,244 (41.1) | 247 (40.4) | 8,734 (41.1) |
| Previous or current, n (%) | 13,222 (58.9) | 364 (59.6) | 12,539 (58.9) |
| **Residential area** |  |  |  |
| Urban, n (%) | 3,367 (15.0) | 77 (12.6) | 3,194 (15.0) |
| Provincial, n (%) | 9,711 (43.2) | 299 (48.9) | 9,162 (43.1) |
| Rural, n (%) | 9,388 (41.8) | 235 (38.5) | 8,917 (41.9) |
| **Road traffic noise levels (dB) at baseline residence** |  |  |  |
| L_den_, mean ± SD | 48.6 ± 8.2 | 53.3 ± 7.4 | 52.7 ± 8.2 |
| LA_24h_, mean ± SD | 52.7 ± 8.2 | 49.2 ± 7.4 | 48.6 ± 8.2 |
| L_d_, mean ± SD | 50.4 ± 8.2 | 51.0 ± 7.5 | 50.4 ± 8.2 |
| L_e_, mean ± SD | 48.1 ± 8.1 | 48.7 ± 7.4 | 48.1 ± 8.1 |
| L_n_, mean ± SD | 44.6 ± 8.0 | 45.1 ± 7.2 | 44.5 ± 8.0 |

Table S2. Association between 24-year mean of road traffic noise L_den_, LA_eq_, L_d_, L_e_, and L_n_, and

incidence of overall breast cancer & breast cancer for women with receptor status in 22,466 nurses

from Danish Nurse Cohort.

| **All breast cancers**  **n = 1,193** | N  cases | Crude Model^a^ | Adjusted Model^b^ |
| --- | --- | --- | --- |
|  |  | HR (95% CI) | HR (95% CI) |
| L_den_, linear per 10 | 1,193 | 1.11 (1.02-1.21) | 1.10 (1.00-1.20) |
| LA_24h_, linear per 10 | 1,193 | 1.11 (1.03-1.21) | 1.10 (1.00-1.21) |
| L_d_, linear per 10 | 1,193 | 1.11 (1.03-1.21) | 1.10 (1.00-1.20) |
| L_e_, linear per 10 | 1,193 | 1.11 (1.03-1.21) | 1.10 (1.00-1.20) |
| L_n_, linear per 10 | 1,193 | 1.11 (1.02-1.21) | 1.09 (0.99-1.20) |
| **Breast cancer with ER & PR status**  **n = 611** |  |  |  |
| L_den_, linear per 10 | 611 | 1.17 (1.04-1.31) | 1.17 (1.02-1.33) |
| LA_24h_, linear per 10 | 611 | 1.17 (1.04-1.31) | 1.16 (1.02-1.33) |
| L_d_, linear per 10 | 611 | 1.16 (1.04-1.30) | 1.16 (1.02-1.32) |
| L_e_, linear per 10 | 611 | 1.17 (1.04-1.31) | 1.17 (1.02-1.33) |
| L_n_, linear per 10 | 611 | 1.17 (1.04-1.32) | 1.17 (1.03-1.34) |

^a^Crude model with age as underlying time scale;
^b^model adjusted for birth cohort, urbanization (urban, provincial, rural), Body Mass Index (underweight, normal,

overweight, obese), leisure time physical activity (low, medium, high), alcohol consumption (low, moderate, heavy),

age at menarche (≤ years of age, > 12 years of age), parity (nulliparous, parous), number of births, age at first birth,

menopausal status (pre-menopausal, post-menopausal), use of hormone therapy (never, past, current), use of oral

contraceptives (never, ever) and smoking status.

Table S3. Association between road traffic noise L_den_ and incidence of breast cancer by ER and PR status in 22,466 nurses from Danish Nurse Cohort.

|  | Breast Cancer Type by ER status | | | | Breast Cancer Type by PR status | | | |
| --- | --- | --- | --- | --- | --- | --- | --- | --- |
|  | N cases | **ER+** | N cases | **ER-** | N cases | **PR+** | N cases | **PR-** |
|  |  | HR (95% CI) |  | HR (95% CI) |  | HR (95% CI) |  | HR (95% CI) |
| **24 years preceding diagnosis** |  |  |  |  |  |  |  |  |
| Linear per 10 dB | 884 | 1.13 (1.02-1.26)* | 177 | 0.97 (0.76-1.23) | 393 | 1.21 (1.02-1.42)* | 218 | 1.10 (0.89-1.37) |
| Low: < 48 dB | 121 | 1.00 | 27 | 1.00 | 45 | 1.00 | 35 | 1.00 |
| Medium: 48-58 dB | 513 | 1.28 (1.04-1.57)* | 100 | 1.01 (0.64-1.57) | 231 | 1.51 (1.08-2.10)* | 121 | 0.99 (0.67-1.47) |
| High: > 58 dB | 250 | 1.33 (1.05-1.69)* | 50 | 1.13 (0.68-1.88) | 117 | 1.66 (1.14-2.40)* | 62 | 1.11 (0.70-1.75) |
| **10 years preceding diagnosis** |  |  |  |  |  |  |  |  |
| Linear per 10 dB | 884 | 1.09 (0.99-1.21) | 177 | 1.00 (0.80-1.24) | 393 | 1.20 (1.03-1.40)* | 218 | 1.13 (0.92-1.37) |
| Low: < 48 dB | 141 | 1.00 | 30 | 1.00 | 51 | 1.00 | 36 | 1.00 |
| Medium: 48-58 dB | 486 | 1.24 (1.02-1.50)* | 102 | 1.10 (0.72-1.68) | 223 | 1.55 (1.13-2.12)* | 120 | 1.13 (0.77-1.67) |
| High: > 58 dB | 257 | 1.29 (1.03-1.61)* | 45 | 0.96 (0.58-1.60) | 119 | 1.65 (1.16-2.35)* | 62 | 1.16 (0.74-1.81) |
| **1 year at the year of diagnosis** |  |  |  |  |  |  |  |  |
| Linear per 10 dB | 884 | 1.07 (0.98-1.18) | 176 | 1.13 (0.91-1.39) | 392 | 1.21 (1.05-1.39)* | 218 | 1.08 (0.90-1.31) |
| Low: < 48 dB | 153 | 1.00 | 27 | 1.00 | 59 | 1.00 | 39 | 1.00 |
| Medium: 48-58 dB | 479 | 1.22 (1.01-1.47)* | 102 | 1.38 (0.89-2.14) | 216 | 1.42 (1.05-1.91)* | 117 | 1.11 (0.76-1.62) |
| High: > 58 dB | 252 | 1.27 (1.02-1.58)* | 47 | 1.28 (0.77-2.14) | 117 | 1.53 (1.09-2.15)* | 62 | 1.18 (0.76-1.82) |

^a^model adjusted for birth cohort, urbanization (urban, provincial, rural), Body Mass Index (underweight, normal, overweight, obese), leisure time physical activity (low, medium, high), alcohol consumption (low, moderate, heavy), age at menarche (≤ years of age, > 12 years of age), parity (nulliparous, parous), number of births, age at first birth, menopausal status (pre-menopausal, post-menopausal), use of hormone therapy (never, past, current), use of oral contraceptives (never, ever) and smoking status; *p-value <0.05.

Table S4. Descriptive statistics at the year of cohort baseline (1993 or 1999) among 21,884 members of the Danish Nurse Cohort, by estrogen and progesterone breast cancer receptor status

|  | **ER+/PR+** | **ER+/PR-** | **PR+/ER-** | **PR-/PR-** | **No breast cancer** |
| --- | --- | --- | --- | --- | --- |
|  | **N = 384** | **N = 110** | **N = 9** | **N = 108** | **N = 21,273** |
| **Age, Mean (SD)** | 52.4 (7.0) | 53.9 (6.6) | 53.3 (5.4) | 53.2 (7.4) | 53.0 (7.9) |
| **Birth cohort** |  |  |  |  |  |
| *<1935, n (%)* | 76 (19.8) | 30 (27.3) | 2 (22.2) | 26 (24.1) | 4,777 (22.5) |
| *1935-1944, n (%)* | 136 (35.4) | 43 (39.1) | 4 (44.4) | 33 (30.6) | 6,432 (30.2) |
| *1945-1949, n (%)* | 80 (20.8) | 24 (21.8) | 3 (33.3) | 26 (24.1) | 4,488 (21.1) |
| *≥ 1950, n (%)* | 92 (24.0) | 13 (11.8) | 0 (0.0) | 23 (21.3) | 5,576 (26.2) |
| **Body Mass Index, mean (SD)** | 23.9 (3.4) | 24.1 (3.9) | 23.1 (2.1) | 23.9 (3.1) | 23.7 (3.5) |
| *BMI <18.5 kg/m^2^, n (%)* | 4 (1.0) | 4 (3.6) | 0 (0.0) | 1 (0.9) | 520 (2.4) |
| *BMI 18.5-24,9 kg/m^2^, n (%)* | 282 (73.4) | 72 (65.5) | 8 (88.9) | 77 (71.3) | 14,618 (68.7) |
| *BMI 25-29.9 kg/m^2^, n (%)* | 72 (18.8) | 25 (22.7) | 1 (11.1) | 26 (24.1) | 4,914 (23.1) |
| *BMI >30 kg/m^2^, n (%)* | 26 (6.8) | 9 (8.2) | 0 (0.0) | 4 (3.7) | 1,221 (5.7) |
| **Physical activity** |  |  |  |  |  |
| *Low, n (%)* | 25 (6.5) | 8 (7.3) | 1 (11.1) | 4 (3.7) | 1,387 (6.5) |
| *Medium, n (%)* | 102 (26.6) | 31 (28.2) | 2 (22.2) | 32 (29.6) | 14,138 (66.5) |
| *High, n (%)* | 257 (66.9) | 71 (64.5) | 6 (66.7) | 72 (66.7) | 5,748 (27.0) |
| **Smoking status** |  |  |  |  |  |
| *Never, n (%)* | 119 (31.0) | 23 (20.9) | 3 (33.3) | 37 (34.3) | 7,535 (35.4) |
| *Previous, n (%)* | 117 (30.5) | 38 (34.5) | 4 (44.4) | 34 (31.5) | 6,545 (30.8) |
| *Current, n (%)* | 148 (38.5) | 49 (44.5) | 2 (22.2) | 37 (34.3) | 7,193 (33.8) |
| **Alcohol consumption** |  |  |  |  |  |
| *Does not drink alcohol, n (%)* | 50 (13.0) | 18 (16.4) | 1 (11.1) | 16 (14.8) | 3,255 (15.3) |
| *Moderate drinker (1-14 drinks/week), n (%)* | 235 (61.2) | 62 (56.4) | 4 (44.4) | 71 (65.7) | 13,220 (62.1) |
| *Heavy drinker (> 14 drinks/week), n (%)* | 99 (25.8) | 30 (27.3) | 4 (44.4) | 21 (19.4) | 4,798 (22.6) |
| **Age at menarche** |  |  |  |  |  |
| *≥* 12*, n (%)* | 98 (25.5) | 28 (25.5) | 2 (22.2) | 31 (28.7) | 5,130 (24.1) |
| <12*, n (%)* | 286 (74.5) | 82 (74.5) | 7 (77.8) | 77 (71.3) | 16,143 (75.9) |
| **Parity** |  |  |  |  |  |
| *Nulliparous, n (%)* | 59 (15.4) | 20 (18.2) | 2 (22.2) | 9 (8.3) | 2,973 (14.0) |
| *Parous, n (%)* | 325 (84.6) | 90 (81.8) | 7 (77.8) | 99 (91.7) | 18,300 (86.0) |
| **Number of births in parous women, mean (SD)** | 2.3 (0.9) | 2.2 (0.72) | 2.4 (0.5) | 2.3 (0.9) | 2.34 (0.9) |
| **Age at first birth, mean (SD)** | 22.1 (10.2) | 21.5 (10.7) | 19.1 (11.4) | 23.6 (8.2) | 22.3 (9.7) |
| **Menopausal status** |  |  |  |  |  |
| *Pre-menopausal, n (%)* | 226 (58.9) | 46 (41.8) | 5 (55.6) | 57 (52.8) | 10,792 (50.7) |
| *Post-menopausal, n (%)* | 158 (41.1) | 64 (58.2) | 4 (44.4) | 51 (47.2) | 10,481 (49.3) |
| **Use of hormone theraphy** |  |  |  |  |  |
| *Never, n (%)* | 254 (66.1) | 65 (59.1) | 2 (22.2) | 69 (63.9) | 15,615 (73.4) |
| *Previous, n (%)* | 31 (8.1) | 13 (11.8) | 2 (22.2) | 11 (10.2) | 2,084 (9.8) |
| *Current, n (%)* | 99 (25.8) | 32 (29.1) | 5 (55.6) | 28 (25.9) | 3,574 (16.8) |
| **Use of oral contraceptives** |  |  |  |  |  |
| *Never, n (%)* | 151 (39.3) | 51 (46.4) | 2 (22.2) | 43 (39.8) | 8,734 (41.1) |
| *Previous or current, n (%)* | 233 (60.7) | 59 (53.6) | 7 (77.8) | 65 (60.2) | 12,539 (58.9) |
| **Night work*** |  |  |  |  |  |
| *No, n (%)* | 310 (97.5%) | 76 (89.4%) | 7 (87.5%) | 80 (93.0%) | 15,725 (94.6%) |
| *Yes, n (%)* | 8 (2.5%) | 9 (10.6%) | 1 (12.5%) | 6 (7.0%) | 900 (5.4%) |
| **Residential area** |  |  |  |  |  |
| *Urban, n (%)* | 49 (12.8) | 18 (16.4) | 1 (11.1) | 9 (8.3) | 3,194 (15.0) |
| *Provincial, n (%)* | 185 (48.2) | 49 (44.5) | 7 (77.8) | 58 (53.7) | 9,162 (43.1) |
| *Rural, n (%)* | 150 (39.1) | 43 (39.1) | 1 (11.1) | 41 (38.0) | 8,917 (41.9) |
| **Road traffic noise levels at baseline residence** |  |  |  |  |  |
| *L_den_, mean (SD)* | 54.2 (7.6) | 54.0 (8.0) | 55.3 (6.8) | 53.5 (7.2) | 52.7 (8.2) |
| *L_24h_, mean (SD)* | 50.2 (7.6) | 49.9 (8.0) | 50.9 (6.7) | 49.5 (7.2) | 48.6 (8.2) |
| *L_day_, mean (SD)* | 52.0 (7.6) | 51.7 (8.0) | 52.7 (6.6) | 51.4 (7.2) | 50.4 (8.2) |
| *L_evening_, mean (SD)* | 49.7 (7.5) | 49.4 (8.0) | 50.5 (6.7) | 49.0 (7.1) | 48.1 (8.1) |
| *L_night_, mean (SD)* | 46.1 (7.3) | 45.8 (7.8) | 47.3 (6.9) | 45.4 (6.9) | 44.5 (8.0) |

* only available for 17,122 nurses who were active in labor force at cohort inclusion

Table S5. Modifications of association^a^ between 24-year mean road traffic noise L_den_ (per 10 dB) and incidence of breast cancer by menopausal status, hormone therapy (HT) use, obesity, night work and residential area in 22,466 nurses from Danish Nurse Cohort.

|  | **All breast cancer**  **N = 1,193** | | **ER-**  **N = 177** | |
| --- | --- | --- | --- | --- |
|  | **N cases** | **HR (95% CI)** | **N cases** | **HR (95% CI)** |
| **Menopausal status** |  |  |  |  |
| Pre-menopausal | 596 | 1.08 (0.94-1.22) | 63 | 0.88 (0.63-1.23) |
| Post-menopausal | 597 | 1.12 (0.98-1.28) | 86 | 1.06 (0.74-1.50) |
| P value for interaction |  | 0.3348 |  | 0.6615 |
| **HT use** |  |  |  |  |
| Never | 774 | 1.08 (0.97-1.22) | 112 | 0.97 (0.72-1.31) |
| Previous | 109 | 0.96 (0.71-1.29) | 17 | 0.72 (0.33-1.55) |
| Current | 310 | 1.20 (0.99-1.45) | 48 | 1.06 (0.65-1.73) |
| P value for interaction |  | 0.2779 |  | 0.7149 |
| **Obesity (BMI >30 kg/m^2^)** |  |  |  |  |
| No | 1,116 | 1.13 (1.02-1.24) | 170 | 0.97 (0.76-1.24) |
| Yes | 77 | 0.79 (0.58-1.07) | 7 | 1.18 (0.39-3.55) |
| P value for interaction |  | 0.1385 |  | 0.8036 |
| **Night work*** |  |  |  |  |
| No | 873 | 1.11 (1.00-1.24) | 130 | 0.99 (0.74-1.31) |
| Yes | 47 | 1.14 (0.72-1.83) | 8 | 0.66 (0.23-1.92) |
| P value for interaction |  | 0.5060 |  | 0.3717 |
| **Residential area** |  |  |  |  |
| Urban | 173 | 1.21 (0.88-1.66) | 21 | 1.14 (0.46-2.81) |
| Provincial | 549 | 1.08 (0.92-1.26) | 94 | 0.64 (0.45-0.92) |
| Rural | 471 | 1.09 (0.96-1.23) | 62 | 1.33 (0.93- 1.89) |
| P value for interaction |  | 0.6036 |  | 0.0146 |

^a^model adjusted for birth cohort, urbanization (urban, provincial, rural), Body Mass Index (underweight, normal, overweight, obese), leisure time physical activity (low, medium, high), alcohol consumption (low, moderate, heavy), age at menarche (≤ years of age, > 12 years of age), parity (nulliparous, parous), number of births, age at first birth, menopausal status (pre-menopausal, post-menopausal), use of hormone therapy (never, past, current), use of oral contraceptives (never, ever) and smoking status; *only available for 17,545 nurses.

Table S6. Association between road traffic noise L_den_ (24-years preceding diagnosis) and incidence of overall breast cancer in nurses from Danish Nurse Cohort with additional adjustments for year of cohort inclusion (1993 or 1999) and average municipality income.

| **All breast cancers**  **n = 1,193** | Crude Model^a^  N = 22,466  N cases = 1,193 | Adjusted Model^b^  N = 22,466  N cases = 1,193 | Adjusted  Model^b^ + year of cohort inclusion  N = 22,466  N cases = 1,193 | Adjusted  Model^b^ + average municipality income at baseline  N = 22,466  N cases = 1,193 |
| --- | --- | --- | --- | --- |
|  | HR (95% CI) | HR (95% CI) | HR (95% CI) | HR (95% CI) |
| Linear per 10 dB | 1.11 (1.02-1.21)* | 1.10 (1.00-1.20)* | 1.10* (1.00 - 1.20) | 1.10* (1.00 - 1.21) |
| Low: < 48 dB | 1.00 | 1.00 | 1.00 | 1.00 |
| Medium: 48-58 dB | 1.24 (1.04-1.47)* | 1.19 (1.00-1.42)* | 1.19* (1.00 - 1.42) | 1.20** (1.00 - 1.43) |
| High: > 58 dB | 1.35 (1.12-1.62)* | 1.30 (1.07-1.60)* | 1.30** (1.06 - 1.60) | 1.31*** (1.07 - 1.60) |
| **Breast cancer with ER & PR status**  **n = 611** | Crude Model^a^  N = 22,466  N cases = 611 | Adjusted Model^b^  N = 22,466  N cases = 611 | Adjusted  Model^b^ + year of cohort inclusion  N = 22,466  N cases = 611 | Adjusted  Model^b^ + average municipality income at baseline  N = 22,466  N cases = 611 |
|  | HR (95% CI) | HR (95% CI) | HR (95% CI) | HR (95% CI) |
| Linear per 10 dB | 1.17* (1.04-1.31) | 1.17* (1.02-1.33) | 1.17** (1.02 - 1.33) | 1.16** (1.02 - 1.33) |
| Low: < 48 dB | 1.00 | 1.00 | 1.00 | 1.00 |
| Medium: 48-58 dB | 1.35* (1.06-1.73) | 1.28* (0.99-1.65) | 1.28* (0.99 - 1.65) | 1.28* (0.99 - 1.64) |
| High: > 58 dB | 1.45* (1.11-1.89) | 1.42 (1.06-1.89) | 1.42** (1.06 - 1.88) | 1.41** (1.06 - 1.88) |

^a^Crude model with age as underlying time scale;
^b^Model adjusted for birth cohort, urbanization (urban, provincial, rural), body mass index (underweight, normal, overweight, obese), leisure time physical activity (low, medium, high), alcohol consumption (low, moderate, heavy), age at menarche (≤ years of age, > 12 years of age), parity (nulliparous, parous), number of births, age at first birth, menopausal status (pre-menopausal, post-menopausal), use of hormone therapy (never, past, current), use of oral contraceptives (never, ever) and smoking status

Table S7. Association between road traffic noise L_den_ (24-years preceding diagnosis) and incidence of overall breast cancer in nurses from Danish Nurse Cohort with additional adjustments for air pollutants.

| **All breast cancers**  **n = 1,193** | Crude Model^a^  N = 22,466  N cases = 1,193 | Adjusted Model^b^  N = 22,466  N cases = 1,193 | Adjusted  Model^b^ on reduced dataset (participants with air pollution data)  N = 21,021 N cases = 1,112 | Adjusted Model^b^ + PM_2.5_ at baseline year  N = 21,021 N cases = 1,112 | Adjusted Model^b^ + NO_x_ at baseline year  N = 21,021 N cases = 1,112 |
| --- | --- | --- | --- | --- | --- |
|  | HR (95% CI) | HR (95% CI) | HR (95% CI) | HR (95% CI) | HR (95% CI) |
| Linear per 10 dB | 1.11 (1.02-1.21)* | 1.10 (1.00-1.20)* | 1.08 (0.98-1.19) | 1.08 (0.98-1.19) | 1.10*(0.99-1.22) |
| Low: < 48 dB | 1.00 | 1.00 | 1.00 | 1.00 | 1.00 |
| Medium: 48-58 dB | 1.24 (1.04-1.47)* | 1.19 (1.00-1.42)* | 1.17* (0.97 - 1.40) | 1.17* (0.98 - 1.40) | 1.17* (0.98 - 1.41) |
| High: > 58 dB | 1.35 (1.12-1.62)* | 1.30 (1.07-1.60)* | 1.26** (1.02 - 1.55) | 1.27** (1.03 - 1.57) | 1.30** (1.05 - 1.61) |
| **Breast cancer with ER & PR status**  **n = 611** | Crude Model^a^  N = 22,466  N cases = 611 | Adjusted Model^b^  N = 22,466  N cases = 611 | Adjusted  Model^b^ on reduced dataset (participants with air pollution data)  N = 21,021  N cases = 576 | Adjusted Model^b^ + PM_2.5_ at baseline year  N = 21,021  N cases = 576 | Adjusted Model^b^ + NO_x_ at baseline year  N = 21,021  N cases = 576 |
|  | HR (95% CI) | HR (95% CI) | HR (95% CI) | HR (95% CI) | HR (95% CI) |
| Linear per 10 dB | 1.17 (1.04-1.31)* | 1.17 (1.02-1.33)* | 1.14* (0.99 - 1.30) | 1.16** (1.01 - 1.33) | 1.19** (1.02 - 1.37) |
| Low: < 48 dB | 1.00 | 1.00 | 1.00 | 1.00 | 1.00 |
| Medium: 48-58 dB | 1.35 (1.06-1.73)* | 1.28 (0.99-1.65) | 1.25* (0.96 - 1.61) | 1.26* (0.97 - 1.63) | 1.26* (0.97 - 1.63) |
| High: > 58 dB | 1.45 (1.11-1.89)* | 1.42 (1.06-1.89)* | 1.35** (1.00 - 1.81) | 1.39** (1.03 - 1.87) | 1.43** (1.05 - 1.94) |

^a^Crude model with age as underlying time scale;
^b^Model adjusted for birth cohort, urbanization (urban, provincial, rural), body mass index (underweight, normal, overweight, obese), leisure time physical activity (low, medium, high), alcohol consumption (low, moderate, heavy), age at menarche (≤ years of age, > 12 years of age), parity (nulliparous, parous), number of births, age at first birth, menopausal status (pre-menopausal, post-menopausal), use of hormone therapy (never, past, current), use of oral contraceptives (never, ever) and smoking status

Table S8. Association between road traffic noise L_den_ and incidence of overall breast cancer in nurses from Danish Nurse Cohort with additional noise categories, defined from quartiles of residential road traffic noise exposure at the year of cohort inclusion.

|  | **All breast cancers**  **n = 1,193** | | **Breast cancer with ER & PR status**  **n = 611** | |
| --- | --- | --- | --- | --- |
| **Road traffic noise** | Crude Model^a^  N = 22,466  N cases = 1,193 | Adjusted Model^b^  N = 22,466  N cases = 1,193 | Crude Model^a^  N = 22,466  N cases = 611 | Adjusted Model^b^  N = 22,466  N cases = 611 |
|  | HR (95% CI) | HR (95% CI) | HR (95% CI) | HR (95% CI) |
| **L_den_ 24-years preceding diagnoses** |  |  |  |  |
| L_den_ < 48.6 dB | 1.00 | 1.00 | 1.00 | 1.00 |
| L_den_ 48.6-53.1 dB | 1.19* (0.99 - 1.42) | 1.15 (0.95 - 1.38) | 1.27* (0.99 - 1.65) | 1.22 (0.94 - 1.58) |
| L_den_ 53.1-58.1 dB | 1.17* (0.98 - 1.39) | 1.12 (0.93 - 1.34) | 1.29** (1.01 - 1.66) | 1.22 (0.94 - 1.58) |
| L_den_ > 58.1 dB | 1.30*** (1.09 - 1.54) | 1.25** (1.03 - 1.52) | 1.41*** (1.10 - 1.80) | 1.38** (1.05 - 1.81) |

^a^Crude model with age as underlying time scale;
^b^Model adjusted for birth cohort, urbanization (urban, provincial, rural), body mass index (underweight, normal, overweight, obese), leisure time physical activity (low, medium, high), alcohol consumption (low, moderate, heavy), age at menarche (≤ years of age, > 12 years of age), parity (nulliparous, parous), number of births, age at first birth, menopausal status (pre-menopausal, post-menopausal), use of hormone therapy (never, past, current), use of oral contraceptives (never, ever) and smoking status

Table S9. Descriptive statistics at the cohort baseline (1993 or 1999) among 1,193 female nurses from the Danish Nurse Cohort with breast cancer status at the end of follow-up.

|  | Breast cancer cases with ER & PR receptor status | Breast cancer cases with no information on ER & PR receptor status |
| --- | --- | --- |
|  | N = 611 | N = 582 |
| **Age, mean ± SD** | 52.8 ± 7.0 | 54.4 ± 8.0 |
| **Birth cohort** |  |  |
| <1935, n (%) | 134 (21.9%) | 156 (26.8%) |
| 1935-1944, n (%) | 216 (35.4%) | 230 (39.5%) |
| 1945-1949, n (%) | 133 (21.8%) | 117 (20.1%) |
| ≥ 1950, n (%) | 128 (20.9%) | 79 (13.6%) |
| **Body Mass Index, mean ± SD** | 23.9 ± 3.4 | 23.8 ± 3.6 |
| BMI <18.5 kg/m^2^, n (%) | 9 (1.5%) | 15 (2.6%) |
| BMI 18.5-24,9 kg/m^2^, n (%) | 439 (71.8%) | 406 (69.8%) |
| BMI 25-29.9 kg/m^2^, n (%) | 124 (20.3%) | 123 (21.1%) |
| BMI >30 , n (%) | 39 (6.4%) | 38 (6.5%) |
| **Physical activity** |  |  |
| Low, n (%) | 38 (6.2%) | 41 (7.0%) |
| Medium, n (%) | 406 (66.4%) | 400 (68.7%) |
| High, n (%) | 167 (27.3%) | 141 (24.2%) |
| **Smoking status** |  |  |
| Never, n (%) | 182 (29.8%) | 190 (32.6%) |
| Previous, n (%) | 193 (31.6%) | 163 (28.0%) |
| Current, n (%) | 236 (38.6%) | 229 (39.3%) |
| **Alcohol consumption, mean ± SD** | 121.5 ± 114.0 | 125.0 ± 135.8 |
| Does not drink alcohol, n (%) | 85 (13.9%) | 104 (17.9%) |
| Moderate drinker (1-14 drinks/week), n (%) | 372 (60.9%) | 317 (54.5%) |
| Heavy drinker (> 14 drinks/week), n (%) | 154 (25.2%) | 161 (27.7%) |
| **Age at menarche (years)** |  |  |
| ≥ 12, n (%) | 159 (26.0%) | 142 (24.4%) |
| <12, n (%) | 452 (74.0%) | 440 (75.6%) |
| **Parity** |  |  |
| Nulliparous, n (%) | 90 (14.7%) | 102 (17.5%) |
| Parous, n (%) | 521 (85.3%) | 480 (82.5%) |
| **Number of births in parous women, mean ± SD** | 2.3 ± 0.8 | 2.4 ± 0.9 |
| **Age at first birth in parous women, mean ± SD** | 26.1 ± 4.1 | 26.4 ± 4.1 |
| **Menopausal status** |  |  |
| Pre-menopausal, n (%) | 334 (54.7%) | 262 (45.0%) |
| Post-menopausal, n (%) | 277 (45.3%) | 320 (55.0%) |
| **Use of hormone therapy (HT)** |  |  |
| Never, n (%) | 390 (63.8%) | 384 (66.0%) |
| Previous, n (%) | 57 (9.3%) | 52 (8.9%) |
| Current, n (%) | 164 (26.8%) | 146 (25.1%) |
| **Use of oral contraceptives** |  |  |
| Never, n (%) | 247 (40.4%) | 263 (45.2%) |
| Previous or current, n (%) | 364 (59.6%) | 319 (54.8%) |
| **Night work** |  |  |
| No, n (%) | 473 (95.2%) | 400 (94.6 %) |
| Yes, n (%) | 24 (4.8%) | 23 (5.4%) |
| **Residential area** |  |  |
| Urban, n (%) | 77 (12.6%) | 96 (16.5%) |
| Provincial, n (%) | 299 (48.9%) | 250 (43.0%) |
| Rural, n (%) | 235 (38.5%) | 236 (40.5%) |
| **Road traffic noise levels (dB) at baseline residence** |  |  |
| L_den_, mean ± SD | 53.3 ± 7.4 | 52.6 ± 8.8 |
| LA_24h_, mean ± SD | 49.2 ± 7.4 | 48.5 ± 8.8 |
| L_d_, mean ± SD | 51.0 ± 7.5 | 50.4 ± 8.9 |
| L_e_, mean ± SD | 48.7 ± 7.4 | 48.0 ± 8.8 |
| L_n_, mean ± SD | 45.1 ± 7.2 | 44.4 ± 8.6 |


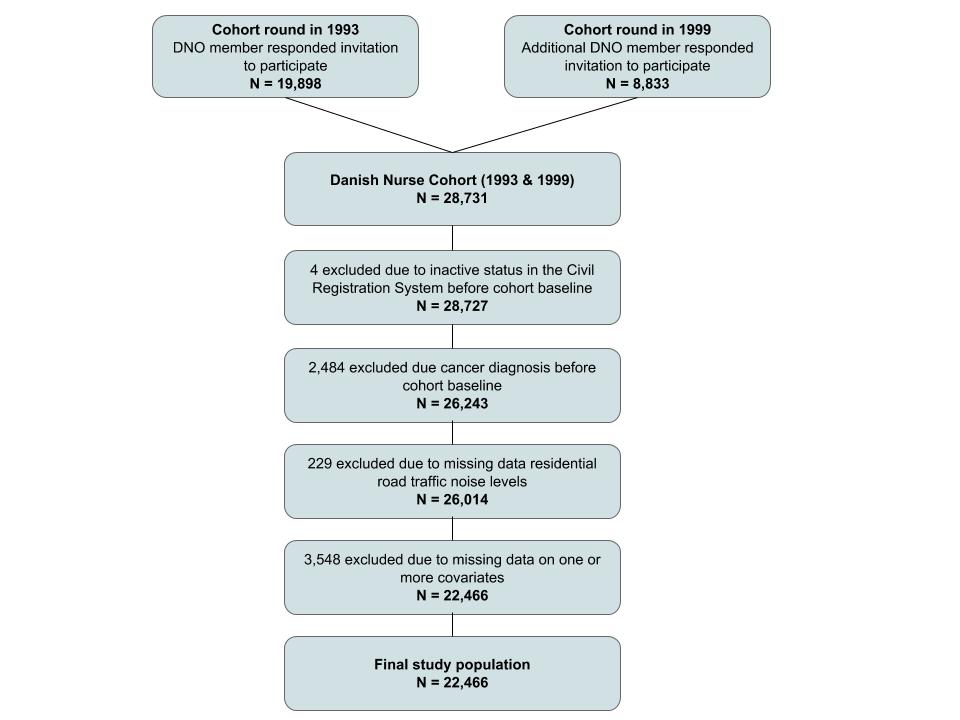
Figure S1. Flow chart over inclusion criteria and study population.
